# Arced Multi-Nozzle Electrospinning Spinneret for High-Throughput Production of Nanofibers

**DOI:** 10.3390/mi11010027

**Published:** 2019-12-24

**Authors:** Jiaxin Jiang, Gaofeng Zheng, Xiang Wang, Wenwang Li, Guoyi Kang, Huatan Chen, Shumin Guo, Juan Liu

**Affiliations:** 1 Department of Instrumental and Electrical Engineering, Xiamen University, Xiamen 361102, China; jiangjx@xmu.edu.cn (J.J.); 35120190154070@stu.xmu.edu.cn (G.K.); 35120190154074@stu.xmu.edu.cn (H.C.); 2 Shenzhen Research Institute of Xiamen University, Shenzhen 518000, China; 3 School of Mechanical and Automotive Engineering, Xiamen University of Technology, Xiamen 361024, China; wx@xmut.edu.cn (X.W.); xmlww@xmut.edu.cn (W.L.); 4 Fujian Provincial Key Laboratory of Mathematical Modeling and High Performance Scientific Computing, School of Mathematical Sciences, Xiamen University, Xiamen 361005, China; shumin_guo@xmu.edu.cn

**Keywords:** multi-jet electrospinning, uniform nanofibers, arced spinneret, electrical field, electrospinning current

## Abstract

The stable and continuous ejection of multiple jets with high densities is the key to the application of electrospinning technology. An arced multi-nozzle spinneret was designed to increase the production efficiency of electrospinning. The distribution of the electrical field was simulated to optimize the nozzles’ distribution of the spinneret. When the nozzles were arranged in an arc array, a relatively uniform electrical field could be obtained, which was beneficial for the weakening of electrical interference among the nozzles. Under the optimized electrical field, multiple jets from each nozzle could be ejected in a stable and continuous way. With the increase of the applied voltage, the electrical stretching force became larger, and there were fewer bonding structures. The average diameter of the electrospun nanofibers decreased with the increase of the applied voltage. When the distance between the inner nozzle and the collector increased, the charged jets suffered a larger stretching effect, resulting in the decrease of the average diameter of the electrospun nanofibers. The electrospinning current increased with the applied voltage and decreased with the distance between the inner nozzle and the collector, which is an important aspect for the monitoring of electrospinning jets. This work provides an effective way to promote the production efficiency of electrospun nanofibrous membranes.

## 1. Introduction

Electrospinning is a typical technology that is used to prepare ultrafine polymer fibers based on the theory of electrohydrodynamics (EHD). Under a high electrical field, the droplets at nozzles are stretched into Taylor cones. When the electrical field force is sufficient to overcome the solution surface tension, thin jets can be ejected from the tips of the Taylor cones. After the high-speed whipping motion and solvent evaporation, a non-woven nanofibrous membrane can be obtained on the grounded collector. Due to their advantages of small diameter, high porosity and large specific surface area, electrospun nanofibers have been widely used in various fields like energy sensing, air filtration, water treatment and biomedicine [1,2,3,4]. However, the low productivity of traditional single-nozzle spinnerets limits the further extension of electrospinning technology [5,6].

In recent years, several multi-jet electrospinning methods have been proposed, including nozzle-less electrospinning and multi-nozzle electrospinning. For nozzle-less electrospinning, without nozzles, the initial position and diameter of jets are random, hindering the uniformity of nanofibrous membranes [7,8,9,10]. For multi-nozzle electrospinning, the distribution of the electrical field strength at different nozzle tips is far from uniform, thus leading to a strong electrical interference among nozzles and making it difficult to realize the stable and continuous ejection of multiple jets [11,12]. A uniform electrical field is the key factor to the stable ejection of multi-nozzle electrospinning. To restrain the distribution of the electrical field, several pieces of equipment have been proposed, including the cylindrical electrode [13], the ring electrode [14], and the plate electrode [15], all of which are helpful in optimizing the uniformity of electrical fields. In addition, the arrangements of nozzles also has a significant effect on the distribution of the electrical field. Yang [16] et al. calculated the electrical field distribution from the electrospinning nozzles to the collector. The differential electrical field of the nozzle array with different heights was much smaller than that with the same height, leading to the uniform distribution of nanofiber diameters.

In this paper, a novel multi-nozzle spinneret is proposed wherein nozzles are arranged in an arc array. The multi-jet ejection behavior and the electrospun nanofibers from the spinneret are investigated. This work provides a simple method for the high-throughput production of uniform nanofibers.

## 2. Materials and Methods

**Figure 1:**
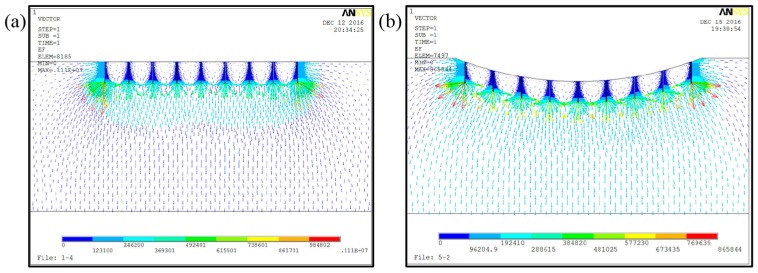
Electrical field simulation of a nozzle array with different arrangements: (**a**) in a linear array and (**b**) in an arc array.

**Figure 2:**
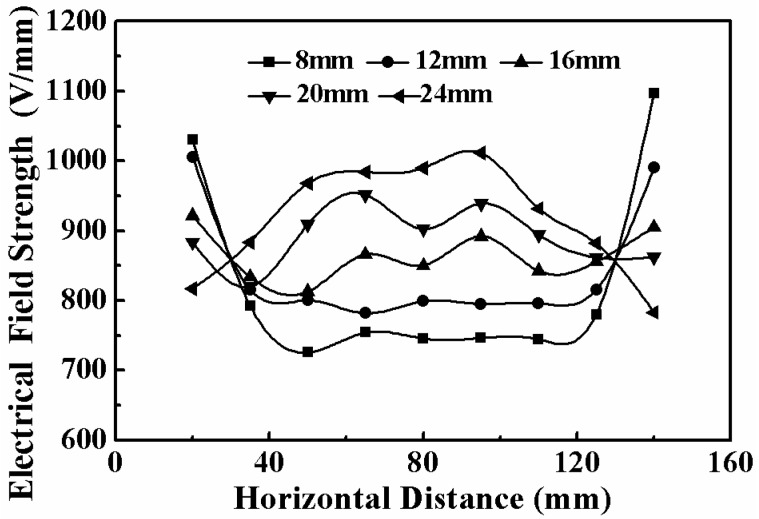
Electrical field strength at nozzle tips with various height differences between the inner nozzle and the outermost nozzle.

The electrical field distribution with different nozzle arrangements was simulated by using the Ansys software first to optimize the spinneret structure, as shown in Figure 1. When the nozzles were arranged in a linear array, the uneven distribution of the electrical field led to strong electrical interference among the nozzles; this interference is called the “end effect” [17]. While the nozzles were arranged in an arc array, the difference of electrical field strength between the outer nozzles and the inner nozzles decreased, and the electrical interference among nozzles was weakened. A uniform electrical field could be beneficial to the formation of Taylor cones and the continuous ejection of jets at each nozzle. The electrical field strength at the nozzle tips in an arc array with different radiuses is depicted in Figure 2. It could be concluded that, with the increase of height difference between the inner nozzle and the outermost nozzle, the electrical field strength at the inner position was gradually enhanced to a level that was even larger than that at the outer position when the height difference was over 16 mm. In this way, a relatively uniform electrical field could be achieved with an optimized nozzle array.

**Figure 3:**
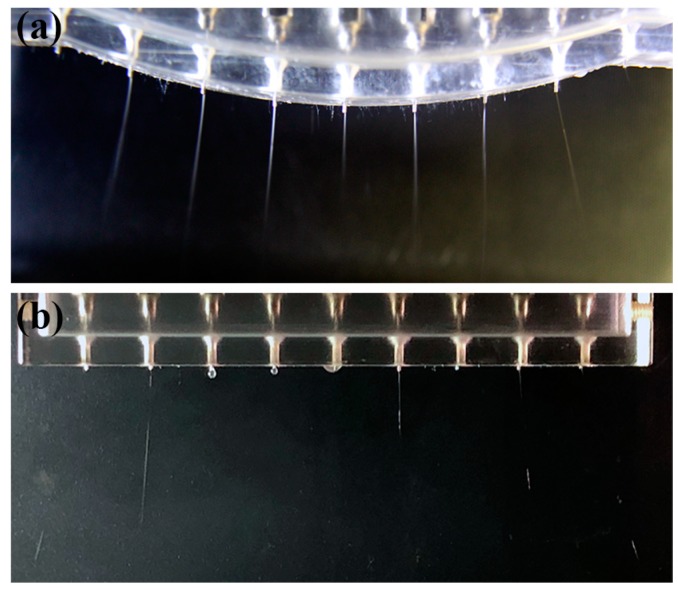
Multi-jet ejection from a multi-nozzle spinneret with different nozzle arrangements: (**a**) in an arc array and (**b**) in a linear array. The applied voltage, the solution concentration, the solution flow rate, and the distance between the inner nozzle and the collector were 12 kV, 8 wt%, 2000 μL/h, and 8 cm, respectively.

Based on the simulation results, a spinneret with 9 nozzles arranged in an arc array was designed, as shown in Figure 3a. Meanwhile, a spinneret arranged in a linear array with the same nozzle number and nozzle distance was designed, as shown in Figure 3b, to verify the influence of electrical field distribution on multi-jet ejection behavior. A high-voltage DC power supply (DW-SA403-1ACE5, Dongwen high voltage power source Ltd., Tianjin, China) was used to generate a high electrical field between the nozzles and the collector. A precision syringe pump (Pump 11 Pico Plus Elite, Harvard Apparatus America, Dover, MA, USA) was used to deliver the electrospinning solution to the nozzles. When the nozzles were arranged in a linear array, the electrical field strength at the inner nozzles was not strong enough to overcome the solution surface tension, so the droplets could not be stretched into Taylor cones and fell straight down to the collector, as shown in Figure 3b. While under the optimized electrical field with the nozzles in an arc array, multiple jets from each nozzle could be ejected in a stable and continuous way, as shown in Figure 3a, thus realizing the high production efficiency of a large-area, electrospun nanofibrous membrane.

The polyethylene oxide (PEO, *M*_w_ = 300,000 g/mol, Changchun Dadi Fine Chemical Co. Ltd., Changchun, China) solution served as the electrospinning solution, of which the powder was dissolved in the solvent mixture of deionized water and absolute ethanol with a volume ratio of 3:1. 

## 3. Results and Discussion

**Figure 4:**
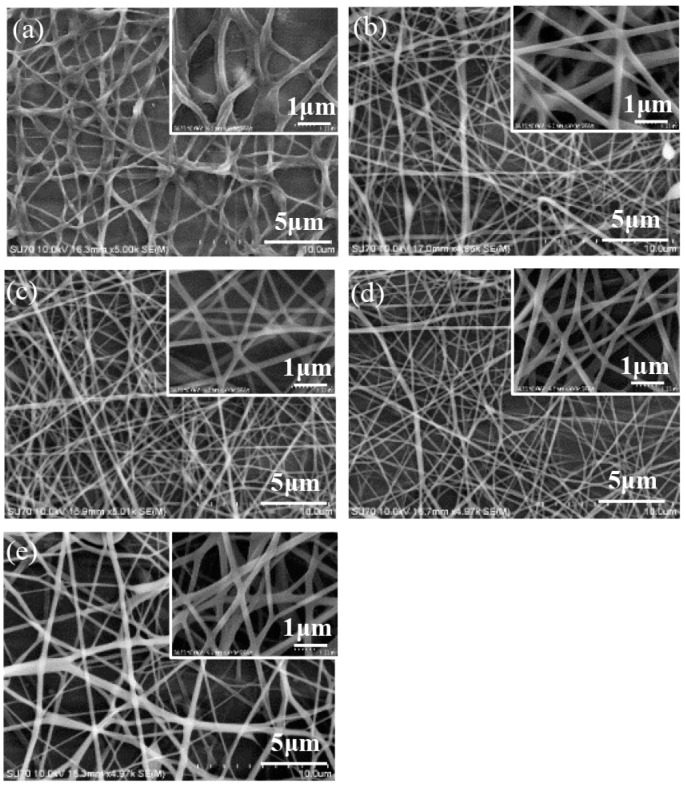
Electrospun nanofibers under different applied voltages: (**a**) 8 kV, (**b**) 10 kV, (**c**) 12 kV, (**d**) 14 kV, and (**e**) 16 kV. The solution concentration, the solution flow rate, and the distance between the inner nozzle and the collector were 8 wt%, 2000 μL/h, and 8 cm, respectively.

**Figure 5:**
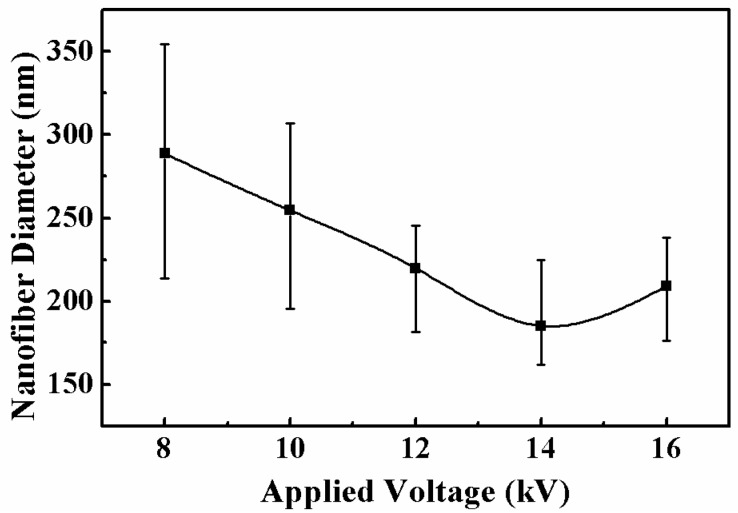
Relationship between nanofiber diameter and applied voltage. The solution concentration, the solution flow rate, and the distance between the inner nozzle and the collector were 8 wt%, 2000 μL/h, and 8 cm, respectively.

The morphology of the electrospun nanofibers from the nozzles in an arc array was investigated. The effect of applied voltage on the nanofibers is presented in Figure 4. When the applied voltage was low, the electrical field force was not strong enough to sufficiently stretch the jets to increase the surface area of the nanofibers; thus, the solvent could not completely evaporate and many bonding structures among the nanofibers existed, as shown in Figure 4a. With the increase of the applied voltage, the electrical stretching force became larger, and there were fewer bonding structures, as shown in Figure 4b–e; thus, the uniformity of the nanofiber diameters was improved. When the applied voltage increased from 8 to 14 kV, the diameter of the electrospun nanofibers decreased from 288.7 ± 74.9 to 185.2 ± 39.8 nm, as depicted in Figure 5. When the applied voltage was 16 kV, the electrical field force was strong enough and the charged jets were fully stretched. The diameter of the nanofibers increased to 209.1 ± 32.7 nm due to the faster speed of jet ejection and the shorter flight time.

**Figure 6:**
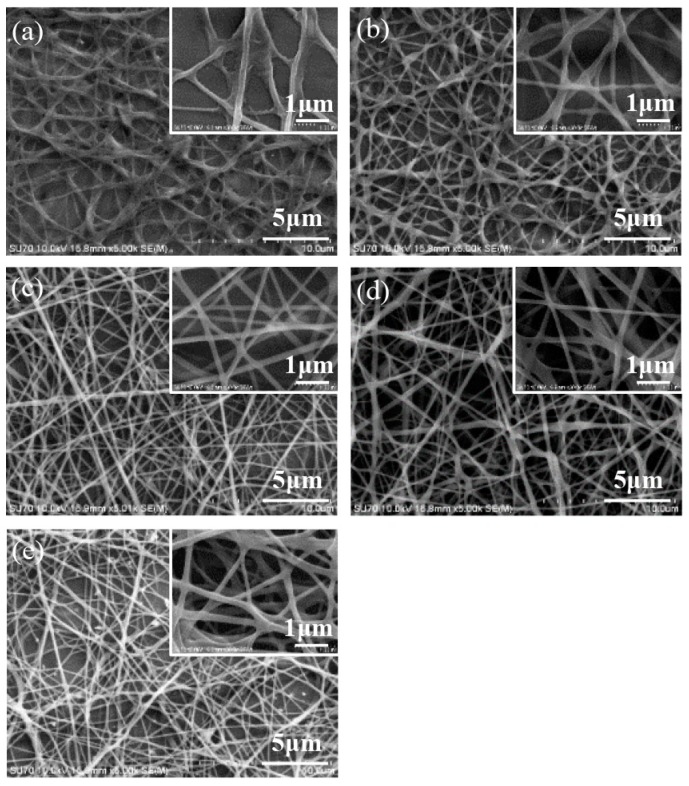
Electrospun nanofibers under different distances between the inner nozzle and the collector: (**a**) 6 cm, (**b**) 7 cm, (**c**) 8 cm, (**d**) 9 cm, and (**e**) 10 cm. The applied voltage, the solution concentration, and the solution flow rate were 12 kV, 8 wt%, and 2000 μL/h, respectively.

**Figure 7:**
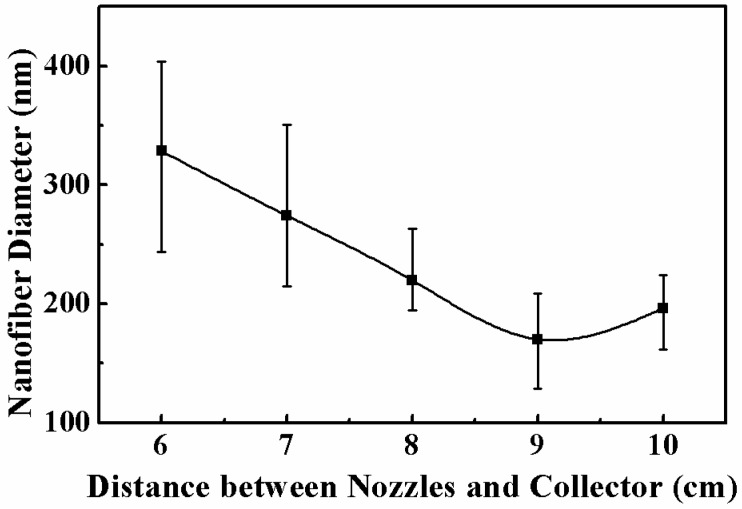
Relationship between nanofiber diameter and the distance between the inner nozzle and the collector. The applied voltage, the solution concentration, and the solution flow rate were 12 kV, 8 wt%, and 2000 μL/h, respectively.

The electrospun nanofibers under different distances between the inner nozzle and the collector are shown in Figure 6. When the distance was shorter than 8 cm, there was not enough time for the solvent to fully evaporate, and some bonding structures existed among the nanofibers, as shown in Figure 6a. With the increase of the distance between the inner nozzle and the collector, the ejecting speed was lower and the flight distance was longer, so the flight time was increased and the solvent could be fully evaporated, as shown in Figure 6b–e; thus, the uniformity of the nanofiber diameters was improved as well. When the distance between the inner nozzle and the collector increased from 6 cm to 9 cm, the charged jets suffered a larger stretching effect, and the diameter of the electrospun nanofibers decreased from 328.6 ± 84.9 to 170.2 ± 41.8 nm, as depicted in Figure 7. When the distance increased to longer than 10 cm, the electrical field force was not strong enough to stretch the jets; thus, the diameter of the nanofibers increased to 196.2 ± 34.5 nm.

**Figure 8:**
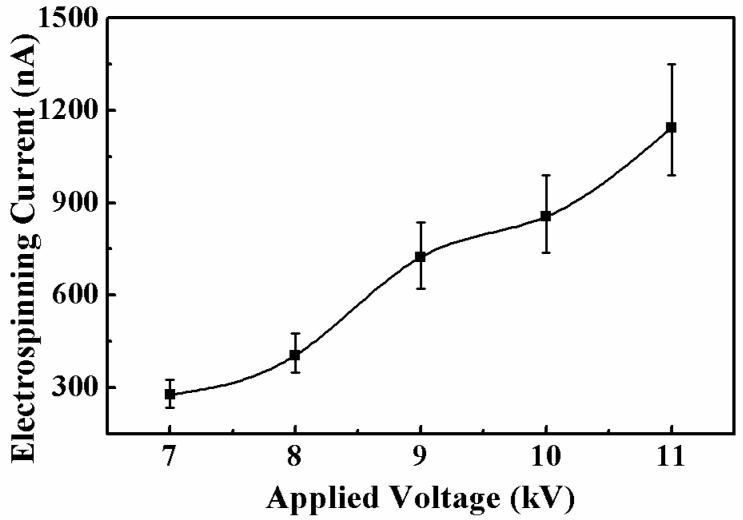
Relationship between the electrospinning current and the applied voltage. The solution concentration, the solution flow rate, and the distance between the inner nozzle and the collector were 8 wt%, 6000 μL/h, and 9 cm, respectively.

**Figure 9:**
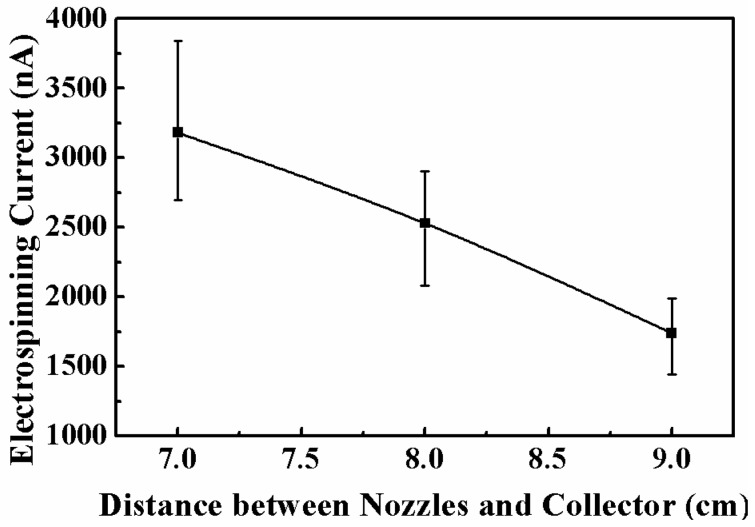
Relationship between the electrospinning current and the distance between the inner nozzle and the collector. The applied voltage, the solution concentration, and the solution flow rate were 10 kV, 10 wt%, and 4000 μL/h, respectively.

With the ejection of multiple jets, a large amount of charges was transferred along the jets driven by the high electrical field, formed the electrospinning current. Thus, the electrospinning current is also an important factor in monitoring the multi-jet electrospinning process. The relationship between the average electrospinning current and the applied voltage is shown in Figure 8. When the applied voltage increased, the electrical field strength and the charge density on the jet surface increased, so the average electrospinning current increased in an approximately linear fashion with the applied voltage. For the applied voltages of 7, 8, 9, 10, and 11 kV, the average electrospinning currents were 276.7, 404.9, 723.8, 855.5, and 1144.4 nA, respectively. Additionally, the effect of the distance between the inner nozzle and the collector on the average electrospinning current was investigated, as depicted in Figure 9. With the increase of the distance between the inner nozzle and the collector, the electrical field strength between the nozzle and the collector decreased, resulting in the decrease of the average electrospinning current. When the distance between the inner nozzle and the collector increased from 7 to 9 cm, the average electrospinning current decreased from 3179.9 to 1738.7 nA.

## 4. Conclusions

A novel spinneret structure was introduced into the process of multi-nozzle electrospinning to achieve the high-throughput production of nanofibers. The electrical field for the multi-nozzle spinneret was simulated, indicating that an optimized array could lead to a uniform distribution of the electrical field, and this uniformity was beneficial to the weakening of electrical interference among the nozzles. Based on the simulation results, a multi-nozzle spinneret, of which the nozzles were arranged in an arc array, was proposed. Multiple jets from each nozzle could be ejected in a stable and continuous way, realizing the high-throughput production of uniform electrospun nanofibrous membranes. The effects of processing parameters on the morphology of nanofibers were investigated. When the applied voltage increased from 8 to 16 kV, the charged jets suffered a larger electrical stretching force and the average diameter of the electrospun nanofibers decreased from 288.7 to 209.1 nm. When the distance between the inner nozzle and the collector increased from 6 to 10 cm, the ejecting speed was lower and the flight distance was longer, so the flight time was increased and the solvent could be fully evaporated; thus, the average diameter of the electrospun nanofibers decreased from 328.6 to 196.2 nm. The electrospinning current was also investigated to characterize the multi-jet electrospinning process. The electrospinning current increased with the applied voltage and decreased with the distance between the inner nozzle and the collector. This work verifies the effectiveness of the novel multi-nozzle spinneret structure in the continuous electrospinning process and, as such, could contribute to the promotion of electrospinning technology in industrial fields.
